# Validation of Artificial Intelligence Computer-Aided Detection of Colonic Neoplasm in Colonoscopy

**DOI:** 10.3390/diagnostics14232762

**Published:** 2024-12-08

**Authors:** Hannah Lee, Jun-Won Chung, Kyoung Oh Kim, Kwang An Kwon, Jung Ho Kim, Sung-Cheol Yun, Sung Woo Jung, Ahmad Sheeraz, Yeong Jun Yoon, Ji Hee Kim, Mohd Azzam Kayasseh

**Affiliations:** 1 Division of Gastroenterology, Department of Internal Medicine, Gachon University Gil Medical Center, Incheon 21565, Republic of Korea; everagape@gmail.com (H.L.); junwonchung@daum.net (J.-W.C.); toptom@gilhospital.com (K.A.K.); junghokimm@gilhospital.com (J.H.K.); 2 Division of Biostatistics, Center for Medical Research and Information, University of Ulsan College of Medicine, Seoul 05505, Republic of Korea; ysch97@amc.seoul.kr; 3 Division of Gastroenterology, Department of Internal Medicine, Korea University College of Medicine, Ansan 15355, Republic of Korea; sungwoojung@korea.ac.kr; 4 CAIMI Co., Ltd., Incheon 22004, Republic of Korea; sheeraz@caimi.co.kr (A.S.); yjyoon@caimi.co.kr (Y.J.Y.); gilgi-jhkim@naver.com (J.H.K.); 5 Division of Gastroenterology, Dr. Sulaiman AI Habib Medical Group, Dubai Healthcare City, Dubai 51431, United Arab Emirates; drmakdxb@gmail.com

**Keywords:** artificial intelligence, computer-aided detection, colonic neoplasm

## Abstract

Background/Objectives: Controlling colonoscopic quality is important in the detection of colon polyps during colonoscopy as it reduces the overall long-term colorectal cancer risk. Artificial intelligence has recently been introduced in various medical fields. In this study, we aimed to validate a previously developed artificial intelligence (AI) computer-aided detection (CADe) algorithm called ALPHAON^®^ and compare outcomes with previous studies that showed that AI outperformed and assisted endoscopists of diverse levels of expertise in detecting colon polyps. Methods: We used the retrospective data of 500 still images, including 100 polyp images and 400 healthy colon images. In addition, we validated the CADe algorithm and compared its diagnostic performance with that of two expert endoscopists and six trainees from Gachon University Gil Medical Center. After a washing-out period of over 2 weeks, endoscopists performed polyp detection on the same dataset with the assistance of ALPHAON^®^. Results: The CADe algorithm presented a high capability in detecting colon polyps, with an accuracy of 0.97 (95% CI: 0.96 to 0.99), sensitivity of 0.91 (95% CI: 0.85 to 0.97), specificity of 0.99 (95% CI: 0.97 to 0.99), and AUC of 0.967. When evaluating and comparing the polyp detection ability of ALPHAON^®^ with that of endoscopists with different levels of expertise (regarding years of endoscopic experience), it was found that ALPHAON^®^ outperformed the experts in accuracy (0.97, 95% CI: 0.96 to 0.99), sensitivity (0.91, 95% CI: 0.85 to 0.97), and specificity (0.99, 95% CI: 0.97 to 0.99). After a washing-out period of over 2 weeks, the overall capability significantly improved for both experts and trainees with the assistance of ALPHAON^®^. Conclusions: The high performance of the CADe algorithm system in colon polyp detection during colonoscopy was verified. The sensitivity of ALPHAON^®^ led to it outperforming the experts, and it demonstrated the ability to enhance the polyp detection ability of both experts and trainees, which suggests a significant possibility of ALPHAON^®^ being able to provide endoscopic assistance.

## 1. Introduction

Colorectal cancer is the third most common malignancy, with 2 million new cases each year worldwide, and it is the second leading cancer-related cause of death globally [1]. The application of colonoscopy as a colorectal cancer screening modality has been considered to prevent cancer by detecting adenomas and serrated lesions with endoscopic removal. The prognosis of colorectal cancer has improved by the use of commonly introduced colonoscopic screening tests, as well as advancements made in cancer management [2,3]. Considering the importance of these tests in detecting and removing precancerous lesions, the quality control of colonoscopies is a rising issue in the current endoscopic field [4,5]. A reduction in interval colorectal cancer risk by up to 3.0% and in mortality rate by up to 5.0% can be achieved with every 1.0% increase in adenoma detection rate (ADR) [5].

Colorectal cancer after colonoscopy is the most important factor in the quality control of colonoscopies. Though there is no single factor that affects the detection of endoscopic characteristics of colorectal cancer after colonoscopy, missing advanced adenomas or serrated lesions is a crucial problem. A reduction in the incidence of interval colorectal cancer could be achieved by enhancing the adenoma detection rate (ADR) by 20–30% and reducing the number of missed lesions [4,5,6]. However, missed detections are inevitable in the clinical field, with 27% depending on non-endoscopic factors such as the bowel preparation scale and the endoscopist’s expertise in detecting precancerous lesions and polyps [7,8]. To improve the endoscopic-dependent factors of colonoscopic quality, various technical advancements have been made in the endoscopic field, including the introduction of chromoendoscopy and high-resolution image techniques [9,10,11].

Currently, artificial intelligence has been applied in various medical fields, including colonoscopic exams [12,13,14,15,16,17,18]. Special attention has been directed to artificial intelligence due to its ability to detect precancerous lesions and improve misdiagnosis [19,20,21]. Deep learning in artificial intelligence enables key information to be taken and learnt from typical features of a large amount of data in the medical field [22,23]. The detection and localization of significant lesions are conducted through computer-aided detection (CADe), whereas diagnosis with differentiation is performed with computer-aided diagnosis (CADx). CADe with automatic polyp detection and CADx with the automatic characterization of polyp histology during colonoscopy are practical applications of machine and deep learning which could enhance the quality of clinical colonoscopy in the medical field. Computer-aided detection (CADe) systems, which can assist endoscopists in detecting colonoscopic lesions, have been studied in diverse clinical trials, evaluating their validity and efficacy in detecting precancerous lesions and enhancing the ADR [24,25].

In this study, we aimed to validate a previously developed artificial intelligence (AI) computer-aided detection (CADe) algorithm (ALPHAON^®^) and evaluate the outcomes of CADe in assisting endoscopists with diverse levels of endoscopic clinical experience in detecting colon polyps.

## 2. Materials and Methods

### 2.1. Study Design and Participants

In this paper, a retrospective, single-center, controlled diagnostic study is presented that was performed at Gachon University Gil Medical Center, Republic of Korea, from 25 October 2023 to 29 March 2024.

**Figure 1:**
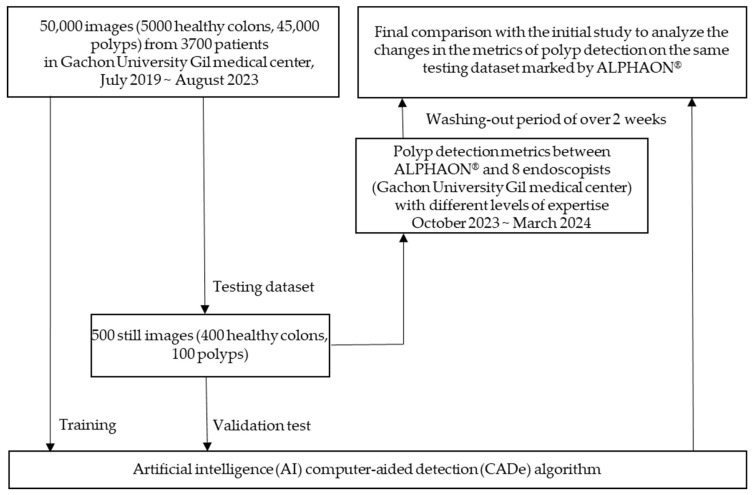
Workflow for development and validation of the artificial intelligence (AI) computer-aided detection (CADe) algorithm.

A retrospectively obtained test dataset of 500 colonoscopic images containing both 400 healthy colon images and 100 polyp images was validated by endoscopists with diverse levels of expertise from Gachon University Gil Medical Center to evaluate the performance of ALPHAON^®^ and generalize its applicability. The overall workflow for the development and validation of the artificial intelligence (AI) computer-aided detection (CADe) algorithm is presented in Figure 1.

Two different methods were used to assess the endoscopic performance of ALPHAON^®^ and the endoscopists in polyp detection.

(1) Colon polyp detection metrics (i.e., accuracy, sensitivity, and specificity) were compared between ALPHAON^®^ and endoscopists across datasets of 500 colonoscopic images containing 400 healthy colon images and 100 polyp images.

(2) After a washing-out period of over 2 weeks, endoscopists performed polyp detection with the same dataset of 500 endoscopic images again with the assistance of ALPHAON^®^. Subsequently, endoscopists utilized ALPHAON^®^’s detection results to make the final assessment and diagnosis. In addition, a final comparison of these results with the initial study was performed to analyze the changes in the metrics. 

### 2.2. Data Preparation and Image Quality Control

A total of 50,000 endoscopic images including 5000 healthy colon images and 45,000 colon polyp images were retrospectively collected from 3700 patients who had undergone colonoscopic examination between July 2019 and August 2023 at Gachon University Gil Medical Center. Colon polyp images were pooled to form regions of interest (ROIs) and utilized to conduct training and validation tests randomly. Each training and test dataset in our study was split ‘per-patient’, ensuring no overlap between the two dataset groups. With the obtained endoscopic datasets, 10-fold cross-validation was utilised to prevent overfitting. The average detection sensitivity was 85% and the error rate per image was 0.04. All endoscopic images were taken and captured in high resolution using different Olympus endoscopes (PCF-H290, CF-H290, CF-HQ290, CF-HQ290Z; Olympus Medical Systems, Tokyo, Japan) with video systems (EVIS LUCERA ELITE CV290/CLV290SL, Olympus Medical Systems).

Endoscopic images of the colonoscopy were stored in a database at Gachon University Gil Medical Center in jpeg format. Only white-light endoscopic images were included, whereas images with narrow-band imaging were not included. In addition, images with poor backgrounds and quality were also excluded. Endoscopists from Gachon University Gil Medical Center with more than 5 years of clinical endoscopic experience that had performed over 5000 endoscopic exams were evaluated regarding the quality of all the images. Colon polyp lesions of the colonoscopic study were all labeled manually. Marking was carefully performed by endoscopists at the border of each polyp lesion. Experienced endoscopists from Gachon University Gil Medical Center performed quality control, labeling, and delineation. Endoscopists in the same group performed labeling and delineation in cooperation, but regarding delineation, one endoscopist processed delineation under the supervision of the other endoscopist. The selection, labeling, and delineation steps were considered completed when the two endoscopists made the same conclusion.

Various recent studies have used convolutional neural networks (CNNs), including Xception, ResNet, and VGG, in colonoscopy work ups to improve polyp detection [26,27,28]. The Xception architecture features a linear stack of depth-wise separable convolution layers with residual connections, which makes the architecture very easy to define and modify. ResNet features an innovative use of residual blocks and is allowed to skip connections, mitigating the vanishing gradient problem during training. This design facilitates the training of very deep networks and enables better visualization. VGG is a deep convolutional neural network with a simple and uniform structure. With a small filter size, it is able to capture fine-grained details. However, apart from CNNs, to enhance polyp detection efficiency, several series of YOLO have been introduced and developed in the endoscopic field. To enhance the efficiency of polyp detection, several target detection algorithms based on the YOLO series have been developed [29,30,31,32]. YOLO models are advanced object detection algorithms combining the Region Proposal Network (RPN) and classification stage into a single network [33]. Nano and small-version YOLO-v10 models were utilized for training in polyp detection systems to identify and categorize polypoid lesions considering its deep learning architecture of high accuracy and efficiency [34]. As the current version, YOLO-v10 especially eliminates the Non-Maximum Suppression (NMS) post-processing step with NMS-free training [34]. A consistent dual-assignment training method is utilized that efficiently reduces the latency. Additionally, YOLO-v10 has made improvements in optimizing the holistic approach to maximize efficiency and accuracy. In addition, it has improved the architecture and capabilities, including changes to the convolutional layers, adding partial self-attention modules to enhance efficiency without risking computational cost [34]. These advancements made in YOLOv10 have been applied to our developed CADe algorithm, which benefits from improved latency for polyp tracking and detection with high suitability.

### 2.3. Validation and Testing of ALPHAON^®^, and Comparison with Endoscopists

The testing images included 500 images of 100 patients from Gachon University Gil Medical Center between July 2019 and August 2023. Endoscopic images were prepared in high resolution using different endoscopes (PCF-H290, CF-H290, CF-HQ290, CF-HQ290Z; Olympus Medical Systems, Tokyo, Japan). The testing dataset included 100 polyps and 400 healthy colon images to compare the performance of ALPHAON^®^ and endoscopists.

A total of 8 endoscopists were recruited from Gachon University Gil Medical Center depending on endoscopic expertise: 2 expert endoscopists and 6 trainees. Endoscopists with experience of more than 15 years were classified as the expert group, whereas 2 endoscopists with an intermediate level of 3 years of endoscopic training and 4 endoscopists in the early stage of their practice (in their first year of endoscopic training) were put in the trainee group.

### 2.4. Statistical Analysis

This study evaluated the performance of a model trained for real-time lesion detection during endoscopy using data from the results of the test dataset of 500 images. It utilized common performance metrics employed in object detection models, including AUC (area under the ROC curve) score, accuracy, sensitivity, and specificity. A statistical confidence interval was computed to estimate the probable range of the true parameter. Logistic regression via generalized estimating equations (GEEs) was applied to evaluate results of the initial validation outcome and washing-out period over 2 weeks later.

The diagnostic validation of ALPHAON^®^ was carried out by evaluating accuracy, sensitivity, and specificity as primary outcomes of this study by calculating the 95% CIs using the Clopper–Pearson method. The receiver operating characteristic (ROC) curve was utilized to demonstrate the diagnostic ability of ALPHAON^®^’s deep learning algorithm in detecting and differentiating patients with colon polyps from the control group. ROC curves were formed by plotting the proportion of true positive cases (sensitivity) against that of false positive cases (1-specificity), by varying the predictive probability threshold for the evaluation of ALPHAON^®^’s diagnostic validation. A better diagnostic performance was indicated by a larger area under the ROC curve (AUC). All statistical tests were two-sided with a significance level of 0.05. Statistical analysis was performed using SPSS (version 26.0; IBM Inc., Armonk, NY, USA).

Accuracy was defined as the case where the deep learning algorithm system identifies a predicted box containing a colon polyp lesion when the confidence value output by ALPHAON^®^ is larger than a given cut-off value. In this study, the cut-off value was defined as the value for which the point on the receiver operating characteristic (ROC) curve has the minimum distance to the upper left corner (where sensitivity is 1 and specificity is 1).

Greater diagnostic accuracy is signified by a higher sensitivity and specificity. This implies that as TPR approaches 1 and FPR approaches 0, the diagnostic accuracy improves. The AUC is essential for a quantitative comparison of the most efficient threshold value.

### 2.5. Outcome Measures

The objective of this study was to evaluate the validity of ALPHAON^®^ in detecting polyps when compared with endoscopists of diverse levels of expertise. The primary and secondary outcomes of this study are listed and categorized as follows.

Co-primary outcomes:

λ Accuracy

Formula:

$$\frac{\left(True\;negative+True\;positive\right)}{\left(True\;negative+False\;positive+False\;negative+True\;positive\right)}$$

$$\mathrm{Accuracy}=\frac{True\;predictions}{Total\;number\;of\;cases}$$

λ Sensitivity

Formula:

$$\frac{True\;positive}{\left(True\;positive+False\;negative\right)}$$

$$\mathrm{Sensitivity}=\frac{True\;positive}{Total\;number\;of\;positive\;cases}$$

λ Specificity

Formula:

$$\frac{True\;negative}{True\;negative+False\;positive}$$

$$\mathrm{Specificity}=\frac{True\;negative}{Total\;number\;of\;negative\;cases}$$

Secondary outcomes:

λ Threshold value;

λ AUC (area under the ROC curve) sensitivity;

λ AUC specificity.

### 2.6. Ethics

The study design was reviewed and approved by the Medical Ethics Committee of Gachon University Medical Center (GCIRB2024-011). The use of retrospectively prepared endoscopic images from the medical center’s database did not require informed consent from participating patients.

## 3. Results

### 3.1. Performance of ALPHAON^®^

**Figure 2:**
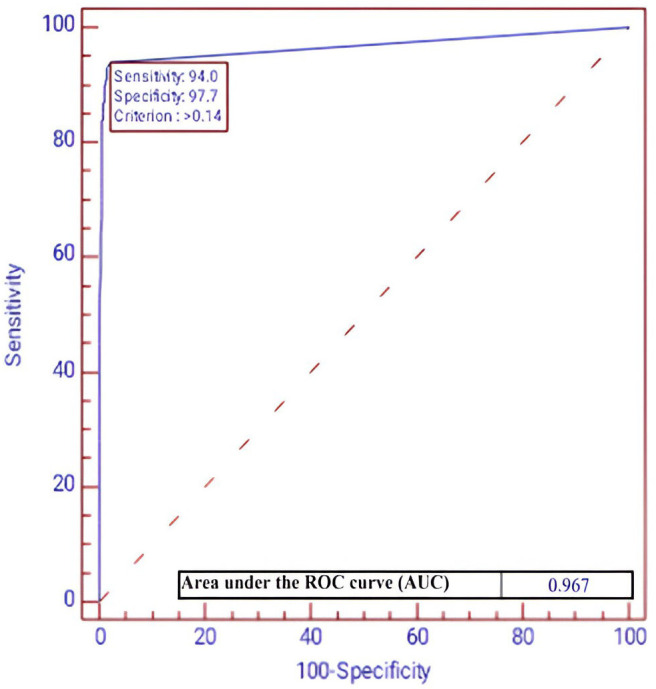
Illustration of ALPHAON^®^’s capability of detecting colon polyps using a receiver operating characteristic curve.

The performance of the CADe algorithm was evaluated by determining its accuracy, sensitivity, specificity, and area under the ROC curve. This algorithm presented a high validity in detecting colon polyps, with an accuracy of 0.97 (95% CI: 0.96 to 0.99), sensitivity of 0.91 (95% CI: 0.85 to 0.97), specificity of 0.99 (95% CI: 0.97 to 0.99) (Table 1), and area under the ROC curve of 0.967 (criterion > 0.14, sensitivity: 0.94, specificity: 0.98) (Figure 2). Evaluating and comparing the polyp detection validity of ALPHAON^®^ with endoscopists with different years of endoscopic experience, ALPHAON^®^ outperformed experts as well as trainees in accuracy (AI: 0.97, 95% CI: 0.96 to 0.99 vs. expert: 0.91, 95% CI: 0.90 to 0.93, *p* < 0.001; trainee: 0.82, 95% CI: 0.79 to 0.85, *p* < 0.001), sensitivity (AI: 0.91, 95% CI: 0.85 to 0.97 vs. expert: 0.71, 95% CI: 0.65 to 0.77, *p* < 0.001; trainee: 0.17, 95% CI: 0.13 to 0.22, *p* < 0.001), and specificity (AI: 0.99, 95% CI: 0.97 to 0.99 vs. expert: 0.97, 95% CI: 0.96 to 0.98, *p* = 0.034; trainee: 0.98, 95% CI: 0.97 to 0.99, *p* = 0.266), but a significant overall validity outcome was present especially in expert endoscopists.

### 3.2. Comparison Between ALPHAON^®^ and Endoscopists

In order to evaluate the practical role of the developed CADe algorithm in the clinical field, we evaluated the polyp detection ability of endoscopists with diverse levels of expertise with and without the assistance of ALPHAON^®^. Given the 2 week washing-out period between the two test steps, independence in polyp detection from the first to second step was assumed. In the first study, the validity according to accuracy, sensitivity, and specificity between three participating groups was evaluated. Participants were categorized into three groups: the expert group, trainee group, and computer-aided detection (CADe) system, ALPHAON^®^ group.

**Figure 3:**
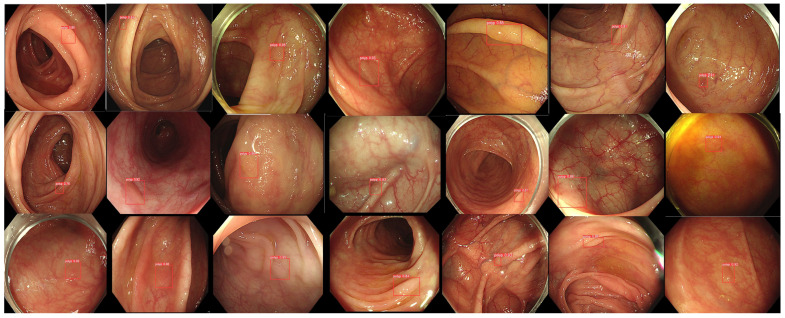
A total of 21 different endoscopic images of colon polyps from testing datasets marked with bounding boxes by CADe algorithm.

**Figure 4:**
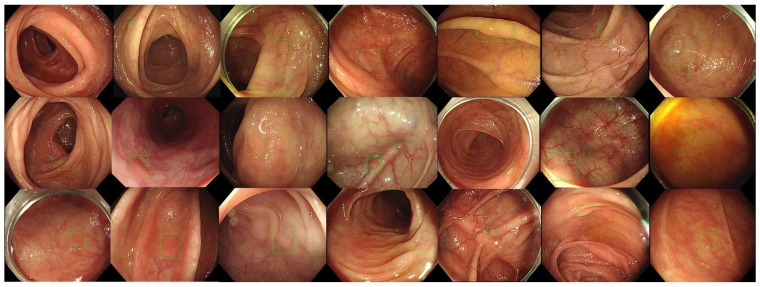
A total of 21 different endoscopic images of colon polyps from testing datasets marked with bounding boxes by expert endoscopists.

**Figure 5:**
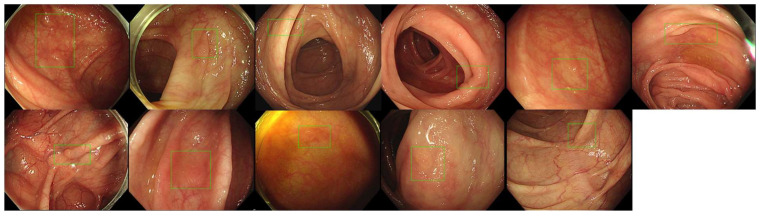
A total of 11 different endoscopic images of colon polyps from testing datasets marked with bounding boxes by trainees, suggesting missing the detection of 10 more polyps compared with AI and experts.

After the 2 week washing-out period, we investigated the follow-up evaluation of and the improvement in the validity of accuracy, sensitivity, and specificity in each participant group with ALPHAON^®^’s assistance. Mixed endoscopic images of 400 healthy colons and 100 colon polyps were presented to endoscopic participants, who were asked to draw bounding boxes around the lesions they expected to be colon polyps with high certainty. Figure 3 presents 21 typical endoscopic images with colon polyps among 100 testing datasets initially marked by each group with bounding boxes. The colon polyp detection results marked with drawn boxes by the CADe algorithm (Figure 3) and experts (Figure 4) correlate with each other, whereas there are missing polyp detections in addition to false positive lesion detections in the trainee results (Figure 5). Comparing the correlation and gap between experts, ALPHAON^®^, and trainees, a high validation of ALPHAON^®^ with experts and a beneficial outcome of the application of ALPHAON^®^ to trainees to progress was expected.

An overall improvement in validity was present in both expert and trainee groups after application of the CADe algorithm. Significant progression with the experts was observed especially in regard to accuracy (0.96, 95% CI: 0.94 to 0.97, *p* < 0.001), sensitivity (0.84, 95% CI: 0.79 to 0.89, *p* < 0.001), and specificity (0.99, 95% CI: 0.97 to 0.99, *p* < 0.001). In the case of trainees, significant improvement was shown in accuracy (0.95, 95% CI: 0.93 to 0.96, *p* < 0.001) and sensitivity (0.80, 95% CI: 0.74 to 0.86, *p* < 0.001) with the assistance of ALPHAON^®^.

## 4. Discussion

Reducing missed colorectal neoplastic lesions and enhancing the adenoma detection rate are important key factors in improving the quality of colonoscopies and reducing interval colorectal cancer rates [35].

This is a retrospective, controlled diagnostic study evaluating the validity and clinical benefit of the newly developed CADe, ALPHAON^®^, in assisting colonoscopies for less experienced junior endoscopists-in-training as well as experts. Our study demonstrated a high ability of ALPHAON^®^, significantly outperforming experts and trainees in accuracy, sensitivity, and specificity (accuracy: 0.97, 95% CI: 0.96 to 0.99; sensitivity: 0.91, 95% CI: 0.85 to 0.97; specificity: 0.99, 95% CI: 0.97 to 0.99). In addition, there was a significant improvement in trainee (accuracy: 0.95 95% CI: 0.93 to 0.96; sensitivity: 0.80 95% CI: 0.74 to 0.86) and expert (accuracy: 0.96, 95% CI: 0.94 to 0.97; sensitivity: 0.84, 95% CI: 0.79 to 0.90) endoscopists in accuracy and sensitivity with the assistance of ALPHAON^®^.

Several recent randomized controlled trials (RCTs) of CADe have shown that CADe improves both the ADR and polyp detection rate when compared with colonoscopies [36,37,38,39]. A high ADR and polyp detection rate were achieved by applying CADe compared to performing conventional colonoscopy alone.

As this retrospective, single-center, and controlled diagnostic study involved testing datasets containing retrospectively gained white-light-cut still images, there were limitations in evaluating the ADR and polyp detection rate. However, the result of our study has not only validated the developed CADe, ALPHAON^®^, but also demonstrated the presence of inter-observer variability during validation, as well as a significant improvement after the application of AI depending on the endoscopists’ expertise.

In another previous study, the variability in the ADR between inter-observers ranged from 7.4 to as high as 52.5% [5]. However, the AI algorithm system can assist in consistently reducing inter-observer variability and achieving the required ADR regardless of the level of expertise or other conflicting factors.

Furthermore, in our study, we confirmed not only the validity of ALPHAON^®^ but also the clinical benefit of CADe in enhancing the detection of polyps both in experts and trainees. Compared with current conventional methods to achieve an improved ADR, such as second forward-view or attachment of a cap, CADe systems are less time consuming and do not feature disposable parts. CADe is a real-time automated conducting system used by endoscopic assistants to detect lesions, guiding the operator in recognizing diminutive mucosal changes and reducing endoscopist fatigue, allowing a reduction in in-day variations in ADRs. These benefits of the CADe system determined by our study highlight the potential of CADe functioning as an educator, particularly for inexperienced trainee endoscopists and in developing countries in need of endoscopic specialists.

Current studies evaluating the performance of CADe-assisted colonoscopy have presented evidence of it supporting trainees and experts in enhancing the rate of adenoma detection [38,40,41,42,43,44,45]. Further clinical trials and studies need to be performed before the widespread introduction of CADe in the endoscopic field.

In spite of these beneficial outcomes and expectations of AI in the clinical field, there are still many aspects that need to be considered and evaluated to improve the current performance of CADe. In addition, there are problems that need to be discussed and considered to generalize the use of CADe in current practice. Questions on cost-effectiveness raised by continuous distractions during the endoscopic procedure [46], the failure of progression in detecting advanced neoplasia [47], the unknown impact on allowing surveillance intervals [48], and the undetermined effect of assisting trainees incapable of solving high false positive detection rates need to be solved and taken into consideration.

The CADe algorithm frequently reports false positive results and may cause fatigue and hardship on the operator during endoscopy. Mucosal tags, bubbles, and folds are commonly detected false positive findings, leading to operator distraction and longer withdrawal times, as well as increased numbers of unnecessary biopsies on lesions [40,41]. In addition, concerns about efficacy and cost-effectiveness are not independent from the frequent false positive issues of AI in the clinical endoscopic area, especially for beginner-level endoscopists who are unable to differentiate true colonic abnormal lesions from false positives.

Optimizing the algorithm and database, and improving sensitivity, which would reduce the false positive rate, would result in maximizing the true benefits and fulfilling the potential of CADe.

Our results have successfully demonstrated the effective role of CADe in assisting both experts and trainees in improving their accuracy and sensitivity significantly, with its performance even outperforming that of experts.

However, there are some limitations in our study. First, regarding testing datasets, we used white-light-cut still images without NBI or video clips, which does not fully reflect an actual endoscopic exam environment. There is a barrier to the application of the results of this study to real lesion detection in colonoscopies due to this dataset, including the results of the evaluation of ADR and polyp detection rate from the testing study step. Future studies with endoscopic datasets including NBI or video clips for data preparation and testing would lead to more productive beneficial outcomes.

Second, our study was performed in a single center with retrospective datasets, which leads to limitations in generalizing the results of testing in the clinical field. Follow-up prospective multicenter studies performed with larger numbers would give more generalized results and supportive evidence for clinical application.

Third, since the study datasets for training and validation are from same center, the presence of an overfitting bias is an inevitable limitation. Future follow-up studies with multicenter datasets would reduce the presence of and dependence on overfitting bias.

Finally, as a CADe system, ALPHAON^®^ has limitations with regard to providing detailed pathological information for the characterization and classification of polyps and colonic lesions, not just detecting and locating the presence of polyps. Therefore, it needs to be further trained and updated to a computer-aided diagnosis (CADx) algorithm with more clinical information, including the pathological result with endoscopic data, to give a more advanced and valuable clinical diagnostic evaluation and more useful information for the current medical environment.

## 5. Conclusions

In this study, the high validation performance of the CADe algorithm system, ALPHAON^®^, in detecting colon polyps was verified, with evidence supporting its outperformance of experts with regard to sensitivity. In addition, the possibility of ALPHAON^®^ being able to assist both experts and trainees in endoscopies to improve sensitivity and specificity was demonstrated. A multicenter prospective validation with refurbished datasets would be required before a generalized introduction and further evaluation of current debates and issues regarding CADe application to the actual clinical endoscopic field can be made.

## Figures and Tables

**Table 1:** Comparison between ALPHAON^®^ and endoscopists with regard to validity in the first and second studies.

	Accuracy (95% CI)	*p*-Value	Sensitivity (95% CI)	*p*-Value	Specificity (95% CI)	*p*-Value
**ALPHAON**	0.97 (0.96 to 0.99)		0.91 (0.85 to 0.97)		0.99 (0.97 to 0.99)	
**Experts**	0.91 (0.90 to 0.93)	<0.001	0.71 (0.65 to 0.77)	<0.001	0.97 (0.96 to 0.98)	0.002
**Trainees**	0.82 (0.79 to 0.85)	<0.001	0.17 (0.12 to 0.22)	<0.001	0.98 (0.97 to 0.99)	0.266
**ALPHAON + experts**	0.96 (0.94 to 0.97)	<0.001	0.84 (0.79 to 0.89)	<0.001	0.99 (0.98 to 0.99)	<0.001
**ALPHAON + trainees**	0.95 (0.93 to 0.96)	<0.001	0.80 (0.74 to 0.86)	<0.001	0.98 (0.97 to 0.99)	0.404

## Data Availability

Since our study is based upon clinical data with retrospective investigation for a diagnostic study, we did not consider registering a repository of the information. Most of the key data findings and results have been presented in the text and raw data supporting the conclusions of our study will be made available by the authors on request.
